# The First Large Deletion of *ATL3* Identified in a Patient Presenting with a Sensory Polyneuropathy

**DOI:** 10.3390/biomedicines11061565

**Published:** 2023-05-28

**Authors:** Ioanna Pyromali, Laurence Richard, Paco Derouault, Jean-Michel Vallat, Karima Ghorab, Corinne Magdelaine, Franck Sturtz, Frédéric Favreau, Anne-Sophie Lia

**Affiliations:** 1 UR 20218, NeurIT, Faculty of Medicine and Pharmacy, University of Limoges, F-87000 Limoges, France; corinne.magdelaine@chu-limoges.fr (C.M.); franck.sturtz@unilim.fr (F.S.); frederic.favreau@unilim.fr (F.F.); anne-sophie.lia@unilim.fr (A.-S.L.); 2 Service de Neurologie, Centre Hospitalier Universitaire (CHU) Limoges, F-87000 Limoges, France; laurence.richard@unilim.fr (L.R.); jean-michel.vallat@chu-limoges.fr (J.-M.V.); karima.ghorab@chu-limoges.fr (K.G.); 3 Service de Bioinformatique, Centre Hospitalier Universitaire (CHU) Limoges, F-87000 Limoges, France; paco.derouault@chu-limoges.fr; 4 Service de Biochimie et de Génétique Moléculaire, Centre Hospitalier Universitaire (CHU) Limoges, F-87000 Limoges, France

**Keywords:** hereditary sensory neuropathy, structural variation, *ATL3*, NGS, CovCopCan

## Abstract

Hereditary sensory neuropathies (HSN) are a heterogenous group of sensory neuropathies. Mutations in *ATL3* have been described in patients presenting with hereditary sensory neuropathy IF (HSN1F), a subtype of HSN. Herein, by analyzing targeted-NGS data of a patient presenting with sensory neuropathy symptoms using the CovCopCan bioinformatic tool, we discovered the presence of a deletion of around 3kb in *ATL3* from Chr11:63,401,422 to Chr11:63,398,182. This deletion affects *ATL3* exons 11 and 12 and could lead to the mutation c.(1036-861_1539+329del), p.(Ala346_Gln513del). In addition, an analysis of the breakpoints’ sequences revealed the presence of Alu transposable elements at the position of the breakpoints, which pointed to a possible erroneous recombination event following a non-allelic-homologous-recombination mechanism in this area. Moreover, electronic microscopy analysis of the patient’s nerve biopsy revealed a severe rarefaction of the myelinated fibers, a demyelinating–remyelinating process, and an abnormal aspect of the endoplasmic reticulum. These findings suggest that this structural variation could potentially be responsible for the HSN symptoms of the patient. Research of structural variations in *ATL3* in numerous other patients presenting similar symptoms should be broadly investigated in order to improve patients’ diagnoses.

## 1. Introduction

Hereditary sensory neuropathies (HSN) are a clinically and genetically heterogenous group of peripheral neuropathies affecting the sensory nerves. Severity and onset of HSN can vary, with patients presenting the first symptoms between the second and fourth decade of life [1]. HSN’s main symptoms include sensory impairment in the lower limbs and slowly progressive loss of multimodal sensation. The important distal sensory loss can lead to chronic ulcerations in feet and sometimes also in hands, resulting in more severe complications like osteomyelitis, tissue infections, and even requiring toe amputations in the most extreme cases [1,2].

HSN can be classified based on the mutated gene that has been associated with the disease and the inheritance mode (autosomal dominant or recessive) [3]. Therefore, for the HSN forms following an autosomal dominant inheritance mode, the following categories of HSN have been described: hereditary sensory and autonomic neuropathy type IA (HSAN1A; OMIM #162400) caused by mutations in *SPTLC1*, hereditary sensory and autonomic neuropathy type IC (HSAN1C; OMIM #613640) caused by mutations in *SPTLC2*, hereditary sensory neuropathy type ID (HSN1D; OMIM #613708) caused by mutations in *ATL1*, hereditary sensory neuropathy type IE (HSN1E; OMIM #614116) caused by mutations in *DNMT1*, hereditary sensory neuropathy type IF (HSN1F; OMIM #615632) caused by mutations in *ATL3*, and hereditary sensory and autonomic neuropathy type VII (HSAN7; OMIM #615548) caused by mutations in *SCN11A*. On the other hand, HSN forms following an autosomal recessive inheritance mode have been described in: hereditary sensory and autonomic neuropathy type IIA (HSAN2A; OMIM #201300) caused by mutations in the HSN2 isoform of *WNK1*, hereditary sensory and autonomic neuropathy type IIB (HSAN2B; OMIM #613115) caused by mutations in *FAM134B*, hereditary sensory neuropathy type IIC (HSN2C; OMIM #614213) caused by mutations in *KIF1A*, hereditary sensory and autonomic neuropathy type IID (HSAN2D; OMIM #243000) caused by mutations in *SCN9A*, hereditary sensory and autonomic neuropathy type III (HSAN3; OMIM #223900) caused by mutations in *ELP1*, hereditary sensory and autonomic neuropathy type IV (HSAN4; OMIM #256800) caused by mutations in *NTRK1*, hereditary sensory and autonomic neuropathy type V (HSAN5; OMIM #608654) caused by mutations in *NGF*, hereditary sensory and autonomic neuropathy type VI (HSAN6; OMIM #614653) caused by mutations in *DST*, and hereditary sensory and autonomic neuropathy type VIII (HSAN8; OMIM #616488) caused by mutations in *PRDM12*. Among these 15 genes involved in HSN, mainly single nucleotide variants (SNVs) have been identified, whereas structural variants (SVs) have rarely been reported. Regarding *ATL3*, to our knowledge, no pathogenic SV has been described in this gene.

*ATL3* is a paralogue of *ATL1*, localizes to chromosome 11 (11q13.1), and encodes a transcript composed of 13 exons to produce a protein named atlastin-3 [4] (ensemble: ENST00000398868.3, UniProtKB-Q6DD88). Atlastin-3 is a member of the atlastin family of endoplasmic-reticulum-shaping membrane-bound GTPases, like its paralogue ATL1 [5]. *ATL3* is more broadly expressed than ATL1, which mainly localizes in the brain. Hence, *ATL3* is expressed not only in central nervous system (CNS) but also in dorsal root ganglia neurons and in peripheral tissues [6]. GTPases form trans-homooligomers in order to connect membranes and mediate homotypic fusion of endoplasmic reticulum (ER) membranes [7]. Atlastin-3 localizes to ER tubules, where it has a role in the biogenesis of the ER tubular network [6,8]. Like the other atlastin proteins, *ATL3* is composed of a large N-terminal cytoplasmic domain with a GTPase activity, two transmembrane helices that attach the protein within the ER membrane, and an amphipathic helix at the C-terminus. ATL proteins facilitate homotypic fusion of the ER membrane by forming homodimers mediated by GTP. The transmembrane domains and the C-terminal tail of ATL are essential for the fusion of membranes, which is likely achieved through the disruption of the membranes to enable the mixing of lipids. When ATLs from opposing membranes come together, they create a strong membrane-tethering effect that ultimately promotes fusion of the two membranes [9]. Accordingly, atlastin-1, encoded by *ATL1*, plays a role in the formation of the tubular ER network in neurons and is necessary for axon elongation during neuronal development [10]. On the other hand, atlastin-3 is accumulated in punctuate structures corresponding to three-way junctions, which represent crossing-points connecting membranous tubules in order to build a polygonal network in the ER [5].

Atlastin-1 and atlastin-3 are two evolutionarily conserved proteins with similar roles in maintaining ER morphology and function. Mutations in both genes, *ATL1* and *ATL3* respectively, coding for these proteins have been implicated in neurological disorders. According to the Human Gene Mutation Database (HGMD) more than a hundred mutations have been reported in *ATL1*, causing either spastic paraplegia 3 (SPG3A; OMIM #182600) or hereditary sensory neuropathy type 1D (HSN1D; OMIM #613708). Among these mutations, missense mutations have mainly been reported. Interestingly one large deletion (deletion of *ATL1* exon 4) has also been identified [11]. Surprisingly, very few mutations have been described in *ATL3* to date. Heterozygous mutations in *ATL3* have been reported to result in hereditary sensory neuropathy type 1F (HSN1F; OMIM #615632) [5]. Two pathogenic mutations, c.575A > G (p.Tyr192Cys) and c.1013G (p.Pro338Arg), in *ATL3* have been reported in German, Spanish, Bosnian, Chinese, and Brazilian families as well and in all of the families, the disease’s inheritance followed an autosomal dominant transmission mode [5,12,13,14]. Patients presenting with mutations in *ATL3* developed signs of sensory neuropathy affecting lower limbs, bone destruction of toes, recurrent foot ulcerations, osteomyelitis, decrease in superficial sensation, and in some cases, absence of tendon reflexes. However, no neuropathy signs were observed in upper limbs. Sensory nerve conduction studies of German patients showed an axonal sensory neuropathy with no motor nerve affection [5]. Moreover, for both Bosnian and Chinese families, patients’ electrophysiologic studies showed decreased amplitude of sensory nerves and slightly reduced sensory nerve conduction velocities, suggesting an axonal degeneration of the distal sensory nerves [12,13]. Recently, a novel nonsense variant (c.16C > T, p.Arg6*) in *ATL3* has been described in two Iranian families presented with distal impairment of sensory function disturbing superficial touch in the soles and absence of any neuropathic symptom in the upper limbs [15].

Herein, by using the CovCopCan software to analyze the targeted-NGS data of a patient presenting with a sensory polyneuropathy [16], we identified the first SV described in *ATL3*. This SV, a deletion of around 3 kb from Chr11:63,401,422 to Chr11:63,398,182, which affected *ATL3* exons 11 and 12, was present in heterozygous state, and could be involved in the disease of the patient. Moreover, upon conducting a more detailed examination of the regions where the breakpoints occurred, the presence of repetitive elements belonging to the Alu family was revealed, suggesting a possible mechanism responsible for this deletion’s appearance. Therefore, we highlight the importance of SV analysis of NGS data in order to improve patients’ diagnoses not only for neuropathies but also for all inherited diseases, and we discuss the pathogenicity of this new variation.

## 2. Materials and Methods

### 2.1. Patient’s Samples

The proband, a 68-year-old man presented with sensory polyneuropathy with no signs of demyelination on the electroneuromyogram (ENMG). After informed consent was obtained, the patient’s peripheral blood was collected into EDTA tubes, and DNA extraction was performed using standard methods (Illustra-DNA-Extraction-kit-BACC3, GEHC). A nerve biopsy of the patient’s sural nerve was performed as well.

### 2.2. Electronic Microscopy

Human sensory nerves were collected during nerve biopsy and analyzed by electronic microscopy in the Neurology and Anatomic Pathology departments at the University Hospital of Limoges. The collected tissues were fixed using 2.5% glutaraldehyde in 0.05 M sodium cacodylate buffer and osmificated for 1 h in 1% osmium tetroxide (OsO4). Subsequently, the nerves were dehydrated using graded acetone and embedded in Epon 812-Araldite. For light microscopy analysis, cross-sections of 1 mm thick were treated with toluidine blue stain and observed using a light microscope. Ultrathin sections were also taken and treated with uranyl acetate and lead citrate staining before being analyzed with a JEOL (1400 flash) electron microscope transmission at 120 keV.

### 2.3. Next Generation Sequencing (NGS) and Bioinformatics Analysis

NGS sequencing was performed using a 93-gene custom panel that was specifically designed to diagnose CMT and related neuropathies (as described in [17]). The library was amplified using the Ion-P1-HiQ-Template-OT2-200 kit (Ampliseq-Custom; Life Technologies, Carlsbad, CA, USA), and the resulting amplified library was sequenced on an Ion–Proton sequencer (Life Technologies, Carlsbad, CA, USA) and aligned with the human reference genome GHCh37. To assess the variants, the NM_015459.5 reference sequence for the *ATL3* gene was used with the aid of Alamut-Visual-Interpretation Software v.2.11 (Interactive-Biosoftware, Rouen, France). GnomAD and gnomAD-SV (https://gnomad.broadinstitute.org/, accessed on 21 September 2022) websites listing genetic variants detected in an asymptomatic population were consulted. Moreover, dbSNP135 (National Center for Biotechnology Information [NCBI], http://www.ncbi.nlm.nih.gov/projects/SNP/, accessed on 8 July 2022) and Clin-Var (www.ncbi.nlm.nih.gov/clinvar, accessed on 8 July 2022) databases were also used.

Following NGS sequencing using the Ion–Proton sequencer, the coverage file generated from the sequencing was analyzed using the CovCopCan software to detect structural variants (SVs), as outlined in reference [16]. The CovCopCan software applies a two-stage correction and normalization algorithm to identify any unbalanced SVs, including CNVs (copy number variants), from the NGS data.

To precisely determine the deletion breakpoints, long-range PCR and Sanger sequencing methods were employed. The Master Mix Phusion Flash (Thermo Fisher Scientific, Waltham, MA, USA) was used for the PCR experiments in combination with the following specific primers (Sigma Aldrich): primer Ex9-F: AAGAGCAGTTACAGGCACTG situated in exon 9 and Ex13-R: AACAACTGCATCCCTCACAG situated in exon 13. Sanger sequencing experiments were performed on PCR products by a walking primer strategy by deploying the Big Dye Terminator Cycle Sequencing Kit v2 (ABI Prism, Applied Biosystems, Waltham, MA, USA). The primers (Sigma Aldrich, Saint-Louis, MO, USA) Int10-F: GACAGAGTCTTGCTCTATTGC in intron 10 and Int12-R: AAGTTGCCAAAGGGGAACAC in intron 12 were used to identify the exact breakpoints.

### 2.4. Cartography

Transposable elements were detected using RepeatMasker software (http://www.repeatmasker.org/cgi-bin/WEBRepeatMasker, accessed on 24 November 2022) with default settings, and the graph was generated with the Gviz package [18].

## 3. Results

### 3.1. Patient’s Phenotype

This study relates the symptoms of a patient who presented with signs of a sensitive polyneuropathy, predominantly in the lower limbs. The patient, a 68-year-old man, had a late onset of the disease (first signs appeared around the age of 66). The neurological examination revealed areflexia in the lower limbs and hypoalesthesia going up to the ankles but no motor deficit. Reflexes were present in the upper limbs. No tremor, no obvious ataxia, and no vestibular or cerebellar syndrome were noticed. He presented with *pes cavus*, difficulties in tightrope walking, bilateral and symmetrical burns, and paresthesia in feet arches. Paresthesia had a progressive installation and progressed upward in the lower limbs not exceeding the knee. The tuning fork test values were 1/8 on the ankles, 3.5/8 on the knees, and 5/8 on the hands. The patient could not achieve a monopodal position, the Romberg’s test was positive but not lateralized, and an absence of osteo-tendinous reflexes was noticed as well. The patient also presented with hypersudation, asthenia, sleep disorders, and erectile dysfunction, but no Sjögren syndrome was observed. No dysmetria was observed on finger–nose and heel–knee maneuvers. The patient said his mother also presented some mild symptoms of neuropathy such as walking problems, but she was not available for clinical and genetic examination.

An electroneuromyogram (ENMG) examination was performed as well. Motor stimulus detection revealed a low motor amplitude on the left fibular and tibular nerves, with normal conduction velocities and moderately delayed F waves in the lower limbs. No conduction block was seen. The ENMG parameters of the upper limbs were normal. Regarding sensory nerves, low amplitude of sural potentials with related velocities was noticed, while the other potentials were normal. Finally, muscle detection with a needle did not show any spontaneous activity at rest and was normal during muscle contraction.

**Figure 1:**
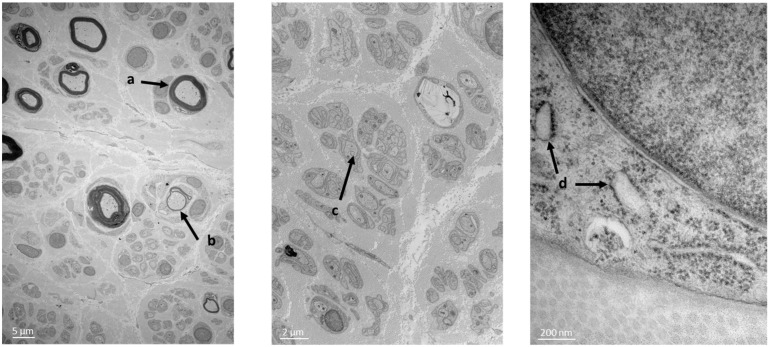
Patient’s nerve biopsy. Semi-fine sections on epon resin revealing rarefaction of the myelinated fibers. Presence of axons with too thin of a myelin sheath in relation to the axonal diameter, supporting a de-myelinating–remyelinating process. (**a**) Normal myelinating fibers; (**b**) de-myelinating–remyelinating fibers; (**c**) unmyelinating fibers; (**d**) ER presenting a suffering morphology.

A sural nerve biopsy was performed. Semi-fine sections on epon resin revealed a very severe rarefaction of the myelinated fibers which appeared to be more or less homogeneous between the fascicles. This damage concerned both large- and small-diameter fibers. A large number of axons with too thin of a myelin sheath in relation to the axonal diameter were present, which indicates a demyelinating–remyelinating process. Regarding the aspect of the endoplasmic reticulum (ER) (Figure 1), it seems that the ER is sometimes dilated without being interconnected, like previously described [9]. No interstitial tissue abnormality was observed.

### 3.2. Structural Variant Detection

**Figure 2:**
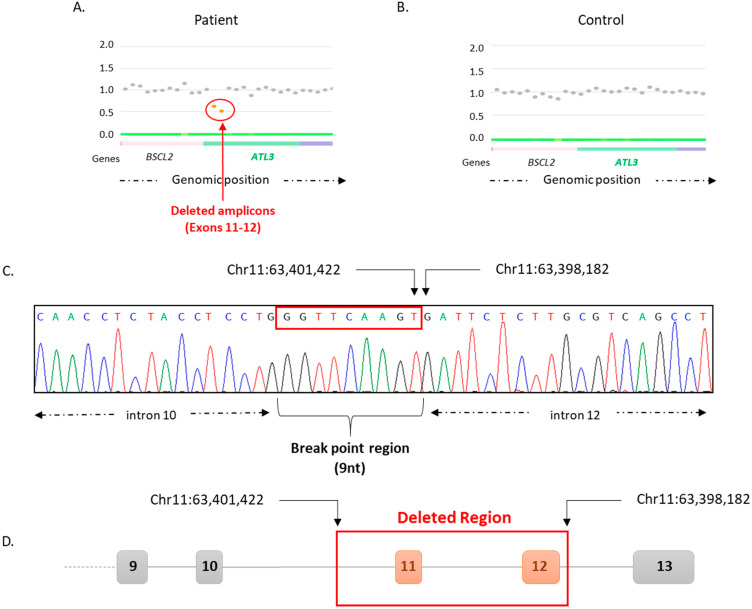
Breakpoint detection on *ATL3* by CovCopCan and Sanger sequencing analysis. CovCopCan graphical representation of the patient (**A**) and a control sample (**B**) on the region of chromosome 11 containing *ATL3* and surrounding genes. Each dot represents an amplicon, and they are distributed along the x-axis according to their genomic position. The y-axis corresponds to the normalized values of each amplicon. Amplicons colored in grey correspond to values around 1, which are considered as normal, whereas amplicons colored in orange correspond to values around 0.5, which are considered as deleted. Two amplicons covering the region of exons 11 and 12 of *ATL3* were revealed as deleted. (**C**) Sanger sequencing results for *ATL3* intron 10 and 12 reveal the deletion’s breakpoints at positions Chr11:63,401,421 and Chr11:63,398,182, with a common region of nine nucleotides (nt) surrounded by the red rectangle. (**D**) Schematic representation of *ATL3* deletion. Non-deleted exons are represented in gray, and deleted exons are represented in orange.

Targeted-NGS by using a panel of 93 genes involved in peripheral neuropathies [17] was employed to analyze the patient’s DNA previously extracted from blood samples. NGS data were then analyzed by the standard alignment bioinformatic analysis, but no positive variant candidate to explain the patient’s phenotype (single nucleotide variant or short indel) was detected. Thus, the user-friendly CovCopCan bioinformatic tool was employed in order to research for any potentially pathogenic structural variant [16]. CovCopCan analysis revealed the deletion of two successive amplicons (Figure 2A,B) corresponding to exons 11 and 12 of *ATL3*, whereas the nearby exons 10 and 13 were not deleted. This deletion covered at least the genomic region Chr11:63,398,506-Chr11:63,400,574 according to the CovCopCan software.

In order to verify the presence of the SV detected by the bioinformatic analysis, a long-range PCR was performed using primers located on the non-deleted exons 9 and 13 of *ATL3*. PCR experiments revealed the presence of a band of around 7000 bp and a second one of around 3700 bp corresponding to the deleted allele and thus confirmed the presence of a deletion in a heterozygous state (data not shown). Then, Sanger sequencing experiments were conducted on the smaller band (corresponding to the deleted allele) in order to identify the exact position of the breakpoints. Thus, the exact breakpoints were identified at Chr11: 63,401,422 in intron 10 at position c.1036-861 and Chr11:63,398,182 in intron 12 at position c.1539+329del, corresponding to a 3240 bp deletion (Figure 2C). This large deletion leads to the variation c.(1036-861_1539+329del), p.(Ala346_Gln513del); which corresponds to the deletion of exons 11 and 12 (Figure 2D).

## 4. Discussion

Inherited genetic disease diagnosis is mainly performed using the targeted next generation sequencing (NGS) technique, followed by a specific bioinformatic analysis allowing detection of pathogenic variants in patients’ samples. Nevertheless, this approach mainly allows detection of point mutations or small indels, while structural variants (SVs) are often underdiagnosed, probably because few user-friendly tools allowing SV detection are available (ExomeDepth, IonCopy, Cov’Cop, DeviCNV, and CovCopCan) [16,19,20,21,22]. Additionally, this type of recently developed software is not being employed systematically in routine analysis of NGS data. In order to improve patients’ diagnoses, we perform SV-research analysis in patients suffering from peripheral neuropathies using the CovCopCan software, and so far, we have detected pathogenic SVs in CMT patients [23,24] and in patients suffering from spastic ataxia of Charlevoix-Saguenay [17]. Moreover, SV research analysis has contributed to improving early diagnosis for patients presenting with inherited peripheral neuropathies [25].

Herein, we analyzed NGS data of a patient presenting with a late-onset sensory polyneuropathy by using the CovCopCan software [16], and we identified the presence of a heterozygous deletion of around 3 kb in *ATL3*. This deletion corresponds to the Chr11:63,401,422-Chr11:63,398,182 genomic positions, affects *ATL3* exons 11 and 12, and potentially leads to the variation c.(1036-861_1539+329del). NGS analysis did not reveal any other possible pathogenic variation that could explain the patient’s symptoms. The patient presented in this study developed typical HSN1F clinical symptoms, such as sensory troubles limited to lower limbs, paresthesia, and altered tendon reflexes, which could be compatible with a variation in *ATL3*, such as those described in previous studies presenting point mutations in *ATL3* [5,12,13,14,15].

*ATL3* codes for atlastin-3, a protein that plays an important role in the biogenesis and structure of the ER tubular network. Electronic microscopy analysis of the sural nerve biopsy of our patient showed abnormal aspects of the patient’s ER, as the ER vesicles appeared more swollen. According to previous studies, a disruption in the ER network has been observed in cells expressing the mutant ATL3, suggesting a dominant-negative effect of mutant *ATL3* [5,9,13]. However, an analysis of the patient’s nerve tissue by using a 3D microscope would be necessary in order to determine the effect of the deletion presented in our patient on the 3D aspects of the ER network.

Homodimerization of *ATL3* of opposing ER membranes promotes tight membrane tethering and eventually membrane fusion, thus contributing to ER dynamics. In the case of the patient described here, the deletion could result in the production of a truncated protein or to the complete absence of the atlastin-3. According to the Alamut Visual Interpretation Software, the deletion described here does not modify the reading frame of the gene. Therefore, provided that RNA splicing is done correctly between exons 10 and 13, we assume that although the protein may be present, the last third of the protein is lacking. If *ATL3* is missing its C-terminal domain but the protein is still able to interact with another monomer including the wild type (WT) version, only 25% of the dimers would be WT–WT and completely active. If the deletion has an effect on the GTP domain or the domain that anchors it to the ER membrane, this means that 75% of the remaining dimers (WT-truncated and truncated-truncated) would have a disturbed activity due to a dominant-negative effect. This kind of dominance mechanism has been observed in other dimeric proteins such as TNSALP involved in hypophosphatasia [26].

**Figure 3:**
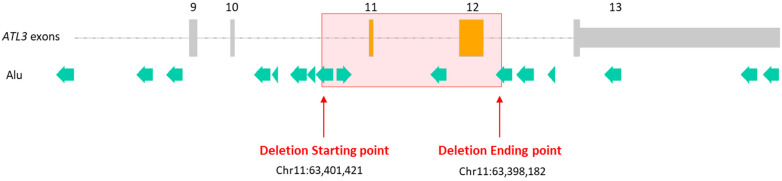
Mapping of *ATL3* structural variation and Alu transposable elements. Non-deleted exons are represented in gray rectangles, and deleted exons are represented in orange rectangles. Green arrows represent transposable elements of the Alu family, and the point of the arrow shows their orientation. The deletion’s breakpoints are situated in a region containing transposable elements of the Alu family, thus promoting a mistaken recombination event.

Once the exact breakpoints were identified, a bioinformatic analysis of the breakpoint regions was conducted using RepeatMasker software. This analysis revealed that in the region of the deletion, there are numerous transposable elements of multiple families. Interestingly, at the exact position where the breakpoints were identified in introns 10 and 12, there is a common sequence of nine identical base pairs (GGTTCAAGT), which corresponds to a transposable element of the Alu transposable element family (Figure 3). The presence of these repetitive elements could lead to an erroneous recombination between two repetitive elements with high sequence identity and the same orientation in the genome and could thus explain the appearance of a deletion in our patient. This genomic rearrangement could occur by the mechanism of non-homologous allelic recombination (NHAR), which is similar to those leading to the well-known *PMP22* structural variations responsible for almost 70% of cases of Charcot–Marie–Tooth disease, for example [27,28,29,30]. This finding leads to the conclusion that the deletion we described for the first time herein could be present not only in the patient described here but in other persons as well. Interestingly, this variation is not present in the GnomAD-SV database, showing its rarity in the general population and suggesting the possible pathogenicity of that variation.

In addition, because of the late onset of the first symptoms in our patient, we infer that the SV described herein could be present in many other patients who have not yet developed symptoms of the disease. Thus, a possible dominant effect of the SV discovered in our study could explain the patient’s phenotype. A dominant-negative effect has been suggested as well for the known missense mutation p.Tyr192Cys, as the mutation caused mislocalization of an endoplasmic-reticulum-shaping GTPase. This mislocalization resulted in axon growth deficits in primary neuron cultures [31].

## 5. Conclusions

To conclude, our approach of analyzing NGS data, not only looking for SNVs but also SVs, allowed us to detect a 3kb deletion in *ATL3* in a patient presenting with sensory neuropathy symptoms. Severe lesions of the peripheral nerve were observed in the patient’s sural nerve biopsy, which were characterized by axonal rarefaction and probable demyelination–remyelination lesions. Further analysis of the breakpoint regions revealed the presence of repetitive elements of the Alu family and suggested a potential mechanism of erroneous homologous recombination that could be at the origin of this deletion. We discussed the possible pathogenicity of this new variation and suggested to search *ATL3* SVs in patients presenting with HSN symptoms in order to improve patients’ diagnoses and be more confident on the pathogenicity of this new variation.

## Data Availability

The data presented in this study are available on request from the corresponding author.
